# Identification of susceptibility pathways for the role of chromosome 15q25.1 in modifying lung cancer risk

**DOI:** 10.1038/s41467-018-05074-y

**Published:** 2018-08-13

**Authors:** Xuemei Ji, Yohan Bossé, Maria Teresa Landi, Jiang Gui, Xiangjun Xiao, David Qian, Philippe Joubert, Maxime Lamontagne, Yafang Li, Ivan Gorlov, Mariella de Biasi, Younghun Han, Olga Gorlova, Rayjean J. Hung, Xifeng Wu, James McKay, Xuchen Zong, Robert Carreras-Torres, David C. Christiani, Neil Caporaso, Mattias Johansson, Geoffrey Liu, Stig E. Bojesen, Loic Le Marchand, Demetrios Albanes, Heike Bickeböller, Melinda C. Aldrich, William S. Bush, Adonina Tardon, Gad Rennert, Chu Chen, M. Dawn Teare, John K. Field, Lambertus A. Kiemeney, Philip Lazarus, Aage Haugen, Stephen Lam, Matthew B. Schabath, Angeline S. Andrew, Hongbing Shen, Yun-Chul Hong, Jian-Min Yuan, Pier A. Bertazzi, Angela C. Pesatori, Yuanqing Ye, Nancy Diao, Li Su, Ruyang Zhang, Yonathan Brhane, Natasha Leighl, Jakob S. Johansen, Anders Mellemgaard, Walid Saliba, Christopher Haiman, Lynne Wilkens, Ana Fernandez-Somoano, Guillermo Fernandez-Tardon, Erik H. F. M. van der Heijden, Jin Hee Kim, Juncheng Dai, Zhibin Hu, Michael P. A. Davies, Michael W. Marcus, Hans Brunnström, Jonas Manjer, Olle Melander, David C. Muller, Kim Overvad, Antonia Trichopoulou, Rosario Tumino, Jennifer Doherty, Gary E. Goodman, Angela Cox, Fiona Taylor, Penella Woll, Irene Brüske, Judith Manz, Thomas Muley, Angela Risch, Albert Rosenberger, Kjell Grankvist, Mikael Johansson, Frances Shepherd, Ming-Sound Tsao, Susanne M. Arnold, Eric B. Haura, Ciprian Bolca, Ivana Holcatova, Vladimir Janout, Milica Kontic, Jolanta Lissowska, Anush Mukeria, Simona Ognjanovic, Tadeusz M. Orlowski, Ghislaine Scelo, Beata Swiatkowska, David Zaridze, Per Bakke, Vidar Skaug, Shanbeh Zienolddiny, Eric J. Duell, Lesley M. Butler, Woon-Puay Koh, Yu-Tang Gao, Richard Houlston, John McLaughlin, Victoria Stevens, David C. Nickle, Ma’en Obeidat, Wim Timens, Bin Zhu, Lei Song, María Soler Artigas, Martin D. Tobin, Louise V. Wain, Fangyi Gu, Jinyoung Byun, Ahsan Kamal, Dakai Zhu, Rachel F. Tyndale, Wei-Qi Wei, Stephen Chanock, Paul Brennan, Christopher I. Amos

**Affiliations:** 1 Biomedical Data Science, Geisel School of Medicine at Dartmouth, Hanover, 03750 NH USA; 2 Department of Molecular Medicine, Laval University, Québec, G1V 4G5 Canada; 3 Institut universitaire de cardiologie et de pneumologie de Québec, Québec, G1V 4G5 Canada; 4 Division of Cancer Epidemiology and Genetics, National Cancer Institute, National Institutes of Health, Bethesda, 20892 MD USA; 5 Annenberg School of Communication, University of Pennsylvania, Philadelphia, 19104 PA USA; 6 Perelman School of Medicine, University of Pennsylvania, Philadelphia, 19104 PA USA; 7 Lunenfeld-Tanenbaum Research Institute, Sinai Health System and University of Toronto, Toronto, M5T 3L9 Canada; 8 Department of Epidemiology, The University of Texas MD Anderson Cancer Center, Houston, 77030 TX USA; 9 International Agency for Research on Cancer, World Health Organization, Lyon, 69372 CEDEX 08 France; 10 Department of Environmental Health, Harvard School of Public Health, Boston, 02115 MA USA; 11 Department of Medicine, Massachusetts General Hospital, Boston, 02115 MA USA; 12 Department of Clinical Biochemistry, Herlev and Gentofte Hospital, Copenhagen University Hospital, Copenhagen, Herlev 2730 Denmark; 13 Faculty of Health and Medical Sciences, University of Copenhagen, Copenhagen, 2200 København N Denmark; 14 Copenhagen General Population Study, Herlev and Gentofte Hospital, Ringvej 75, Copenhagen, Herlev 2730 Denmark; 15 Epidemiology Program, University of Hawaii Cancer Center, Honolulu, 96813 HI USA; 16 Department of Genetic Epidemiology, University Medical Center, Georg-August-University Göttingen, Göttingen, 37073 Germany; 17 Department of Thoracic Surgery, Division of Epidemiology, Vanderbilt University Medical Center, Nashville, 37203 TN USA; 18 Department of Epidemiology and Biostatistics, School of Medicine, Case Western Reserve University, Cleveland, 44106 OH USA; 19 Faculty of Medicine, University of Oviedo, Oviedo, 33006 Spain; 20 Centro de Investigación Biomédica en Red de Epidemiología y Salud Pública, Campus del Cristo s/n, Oviedo, 33006 Spain; 21 Clalit National Cancer Control Center, Carmel Medical Center, Haifa, 34361 Israel; 22 Faculty of Medicine, Technion, Haifa, 34361 Israel; 23 Public Health Sciences Division, Fred Hutchinson Cancer Research Center, Seattle, 98109 WA USA; 24 School of Health and Related Research, University of Sheffield, Sheffield, S1 4DA UK; 25 Roy Castle Lung Cancer Research Programme, Institute of Translational Medicine, University of Liverpool, Liverpool, L69 3BX UK; 26 Radboud University Medical Center, Radboud Institute for Health Sciences, Nijmegen, 6525 EZ The Netherlands; 27 Department of Pharmaceutical Sciences, College of Pharmacy, Washington State University, Spokane, 99210-1495 WA USA; 28 National Institute of Occupational Health, 0033, Gydas vei 8, 0033, Oslo, Norway; 29 British Columbia Cancer Agency, 675 West 10th Avenue, Vancouver, V5Z1L3 Canada; 30 Department of Cancer Epidemiology, H. Lee Moffitt Cancer Center and Research Institute, Tampa, 33612 FL USA; 31 Department of Epidemiology, Geisel School of Medicine, 1 Medical Center Drive, Hanover, 03755 NH USA; 32 Department of Epidemiology and Biostatistics, Jiangsu Key Lab of Cancer Biomarkers, Prevention and Treatment, Collaborative Innovation Center for Cancer Personalized Medicine, School of Public Health, Nanjing Medical University, 101 Longmian Ave, Nanjing, 211166 PR China; 33 Department of Preventive Medicine, Seoul National University College of Medicine, 1 Gwanak-ro, Gwanak-gu, Seoul, 151 742 Republic of Korea; 34 University of Pittsburgh Cancer Institute, Pittsburgh, 15232 PA USA; 35 Department of Preventive Medicine, IRCCS Foundation Ca’Granda Ospedale Maggiore Policlinico, Milan, 20133 Italy; 36 Department of Clinical Sciences and Community Health, University of Milan, Milan, 20133 Italy; 37 University Health Network—The Princess Margaret Cancer Centre, 600 University Avenue, Toronto, M5G 2C4 Canada; 38 Department of Oncology, Herlev and Gentofte Hospital, Copenhagen University Hospital, Copenhagen, 2730 Denmark; 39 Department of Preventive Medicine, Keck School of Medicine, University of Southern California Norris Comprehensive Cancer Center, Los Angeles, 90033 CA USA; 40 Department of Integrative Bioscience & Biotechnology, Sejong University, Gwangjin-gu, Seoul, 05029 Republic of Korea; 41 Department of Pathology, Lund University, Lund, 222 41 Sweden; 42 Faculty of Medicine, Lund University, Lund, 22100 Sweden; 43 School of Public Health, St Mary’s Campus, Imperial College London, London, W2 1PG UK; 44 Hellenic Health Foundation, Athens, GR-115 27 Greece; 45 Cancer Registry and Histopathology Department, “Civic-M.P. Arezzo” Hospital, ASP, Ragusa, 97100 Italy; 46 Fred Hutchinson Cancer Research Center, Seattle, 98109-1024 WA USA; 47 Swedish Medical Group, Arnold Pavilion, Suite 200, Seattle, 98104 WA USA; 48 Department of Oncology and Metabolism, University of Sheffield, Sheffield, S10 2RX UK; 49 Research Unit of Molecular Epidemiology, Institute of Epidemiology II, Helmholtz Zentrum München, German Research Center for Environmental Health, Neuherberg, D-85764 Germany; 50 Thoraxklinik at University Hospital Heidelberg, Heidelberg, 69126 Germany; 51 Translational Lung Research Center Heidelberg (TLRC-H), Heidelberg, 69120 Germany; 52 Cancer Cluster Salzburg, University of Salzburg, Salzburg, 5020 Austria; 53 Department of Medical Biosciences, Umeå University, Umeå, 901 85 Sweden; 54 Department of Radiation Sciences, Umeå University, Umeå, 901 85 Sweden; 55 Princess Margaret Cancer Centre, Toronto, M5G2M9 Canada; 56 Markey Cancer Center, University of Kentucky, First Floor, 800 Rose Street, Lexington, 40508 KY USA; 57 Department of Thoracic Oncology, H. Lee Moffitt Cancer Center and Research Institute, Tampa, 33612 KY USA; 58 Institute of Pneumology “Marius Nasta”, Bucharest, RO-050159 Romania; 59 1st Faculty of Medicine, Charles University, Kateřinská 32, Prague, 121 08 Praha 2 Czech Republic; 60 Clinical Center of Serbia, Clinic for Pulmonology, School of Medicine, University of Belgrade, Belgrade, 11000 Serbia; 61 Department of Cancer Epidemiology and Prevention, M. Sklodowska-Curie Institute—Oncology Center, Warsaw, 02-781 Poland; 62 Department of Epidemiology and Prevention, Russian N.N. Blokhin Cancer Research Centre, Moscow, 115478 Russian Federation; 63 International Organization for Cancer Prevention and Research, Belgrade, 11070 Serbia; 64 Department of Surgery, National Tuberculosis and Lung Diseases Research Institute, Warsaw, PL-01-138 Poland; 65 Department of Environmental Epidemiology, Nofer Institute of Occupational Medicine, Lodz, 91-348 Poland; 66 Department of Clinical Science, University of Bergen, Bergen, 5021 Norway; 67 Unit of Nutrition and Cancer, Catalan Institute of Oncology (ICO-IDIBELL), Barcelona, 08908 Spain; 68 Duke–NUS Medical School, Singapore, 119077 Singapore; 69 Saw Swee Hock School of Public Health, National University of Singapore, Singapore, 117549 Singapore; 70 Department of Epidemiology, Shanghai Cancer Institute, Shanghai, 2200 China; 71 The Institute of Cancer Research, London, SW7 3RP England UK; 72 Public Health Ontario, Windsor, Ontario, N8W 5K5 Canada; 73 American Cancer Society, Inc., Atlanta, 30303 GA USA; 74 Department of Genetics and Pharmacogenomics, Merck Research Laboratories, Boston, 02115-5727 MA USA; 75 Centre for Heart Lung Innovation, St Paul’s Hospital, The University of British Columbia, Vancouver, V6Z 1Y6 BC Canada; 76 Department of Pathology and Medical Biology, GRIAC, University of Groningen, University Medical Center Groningen, Groningen, NL - 9713 GZ The Netherlands; 77 Genetic Epidemiology Group, Department of Health Sciences, University of Leicester, Leicester, LE1 7RH UK; 78 Leicester Respiratory Biomedical Research Unit, National Institute for Health Research (NIHR), Glenfield Hospital, Leicester, LE3 9QP UK; 79 Department of Pharmacology and Toxicology, University of Toronto, Toronto, M5S 1A8 ON Canada; 80 Department of Psychiatry, University of Toronto, Toronto, M5T 1R8 ON Canada; 81 Campbell Family Mental Health Research Institute, Centre for Addiction and Mental Health, Toronto, M6J 1H4 ON Canada; 82 Department of Biomedical Informatics, School of Medicine, Vanderbilt University, Nashville, TN 37235 USA; 83 The Institute for Clinical and Translational Research, Baylor College of Medicine, Houston, 77030 TX USA

## Abstract

Genome-wide association studies (GWAS) identified the chromosome 15q25.1 locus as a leading susceptibility region for lung cancer. However, the pathogenic pathways, through which susceptibility SNPs within chromosome 15q25.1 affects lung cancer risk, have not been explored. We analyzed three cohorts with GWAS data consisting 42,901 individuals and lung expression quantitative trait loci (eQTL) data on 409 individuals to identify and validate the underlying pathways and to investigate the combined effect of genes from the identified susceptibility pathways. The KEGG neuroactive ligand receptor interaction pathway, two Reactome pathways, and 22 Gene Ontology terms were identified and replicated to be significantly associated with lung cancer risk, with *P* values less than 0.05 and FDR less than 0.1. Functional annotation of eQTL analysis results showed that the neuroactive ligand receptor interaction pathway and gated channel activity were involved in lung cancer risk. These pathways provide important insights for the etiology of lung cancer.

## Abstract (web summary)

The chromosome 15q25.1 locus is a leading susceptibility region for lung cancer. Here, the authors interrogate three GWAS cohorts with 42,901 individuals to investigate potential pathological pathways such as gated channel activity and neuroactive ligand receptor interaction in lung cancer etiology.

## Introduction

Lung cancer, accounting for 13% of all cancer cases and 23% of all cancer-related deaths worldwide, is a leading cause of cancer death in the US and around the world, and represents a major public health problem^1^. Several genome-wide association studies (GWAS) have been published and identified the chromosome 15q25.1 locus as a susceptibility region for lung cancer^2–4^, smoking behavior^5,6^, and nicotine addiction^4^ in Caucasians^2^, African-Americans^7^, and Asians^8^. Epigenetic analyses provided evidence that epigenetic silencing of nAChR-encoding genes clustered at the 15q25.1 locus may contribute to lung cancer risk^9^. In addition, expression quantitative trait loci (eQTL) studies showed an influence of alleles in this region on the expression of several genes at chromosome 15q25.1, providing a mechanism by which these variations might affect lung cancer risk^10^. Our previous GWA studies found that variants in chromosome 15q25.1, including single-nucleotide polymorphisms (SNPs) and haplotypes, are involved in the etiology of overall lung cancer susceptibility and by histology and smoking status^2,11^. However, lung cancer, being a disease of complex origin, is usually considered to result from complex effects of smoking along with multiple genetic variants affecting a number of pathways or biological process. Common SNPs are not individually known to add greatly to individual risk, unless more complex gene–gene interactions play a crucial role in the genetic architecture of pathogenesis of complex disorders^12^, such as lung cancer. The pathogenic pathways, through which lung cancer susceptibility SNPs within chromosome 15q25.1 affect disease etiology and development of lung cancer, have not been studied comprehensively, limiting mechanistic understanding.

The objective of this study was to explore the underlying pathways that are involved in the molecular mechanisms by which variants at the chromosome 15q25.1 locus modify lung cancer risk and increase lung cancer occurrence and development. We first performed a GWAS analysis with a cohort of 1923 lung cancer cases and 1977 healthy controls of Italian origin combined with a cohort of 2995 lung cases and 3578 controls of European ancestry, and then conducted a meta-analysis to identify the index SNPs within the chromosome 15q25.1 locus that were significantly associated with lung cancer risk. We then investigated the SNP–SNP interaction between the index SNPs within the chromosome 15q25.1 locus and the entire genome to identify the SNPs that interact with the 15q25.1 index SNPs, and are therefore involved in lung cancer etiology through interaction. Furthermore, using the index SNPs and their related SNPs in the whole genome, we explored the pathogenic pathways that may be relevant to lung cancer etiology, and replicated the findings with an independent cohort of 18,439 lung cancer cases and 14,026 healthy controls. We also studied genome-wide gene expression data in human lung tissues and conducted an eQTL analysis to investigate whether the functional annotation of the eQTL results can validate the susceptibility pathways from our GWAS analyses. Finally, we explored whether genes from our susceptibility pathways might jointly affect the process by which the chromosome 15q25.1 locus influences lung cancer risk. Our findings suggest that common genetic variations within chromosome 15q25.1 are likely to affect lung cancer etiology by influencing the expression/structure and thereby the function of genes that comprise the neuroactive ligand receptor interaction pathway or gated channel activity and related terms. Such new biologic insights from pathway analysis will provide a better understanding of the etiology and development of lung cancer, potentially shortening the interval between increasing biologic knowledge and translation to patient care.

## Results

**Fig. 1:**
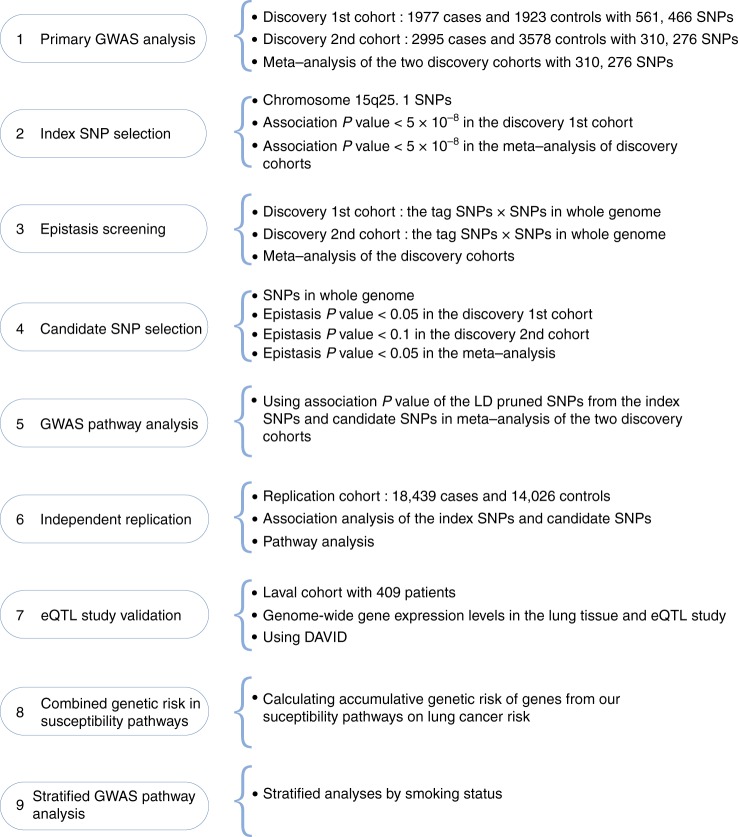
Schematic overview of the study design. (1) In the discovery phase, a total of 310,276 SNPs were the same in both the 1st and 2nd discovery cohorts and were applied for association analyses and meta-analyses. (2) SNPs within the 15q25.1 locus, which were associated with lung cancer risk with logistic regression *P* values of less than 5 × 10^−8^ in the 1st discovery cohort and in the meta-analysis of the discovery cohorts, were selected as index SNPs. (3) The epistasis test between SNPs in the whole genome and the index SNPs within chromosome 15q25.1 locus were conducted for both discovery cohorts and a meta-analysis was performed to combine the epistasis results. (4) The SNPs, which interacted with the index SNPs with an epistasis *P* value of less than 0.05 in the 1st discovery cohort and in the meta-analysis of both discovery cohorts, and less than 0.10 in the 2nd discovery cohort, were selected as the candidate SNPs. (5) The index SNPs and the candidate SNPs with the logistic regression *P* values in the meta-analysis of discovery cohorts were applied for GWAS pathway analysis. (6) In the replication phase, the index SNPs and the candidate SNPs with the logistic regression *P* values in an independent cohort were applied for GWAS pathway analysis to validate the susceptibility pathway enriched in step **5**. (7) The most significant genes in the whole genome regulated by SNPs in chromosome 15q25.1, which were selected with the eQTL study, were employed for pathway analysis. (8) The individual and combined effects of genes in the pathways on lung cancer risk were calculated. (9) A similar process to select index SNPs and candidate SNPs and to carry out GWAS pathway analyses in the subgroups of smokers and non-smokers were conducted

The study design is presented in Fig. 1. Demographic characteristics and sample sizes of the two discovery cohorts and the replication cohort for GWAS pathway analyses are summarized in Table 1. Demographic characteristics of the lung eQTL study are summarized in Supplementary Table 1.

**Table 1:** Participant characteristics of lung cancer cases and controls in GWAS cohorts

Variants	1st Discovery cohort (*n* = 3900)					2nd Discovery cohort (*n* = 6573)					Replication Cohort (*n* = 32,465)				
	Control (*n* = 1977)		Case (*n* = 1923)		*P*-value	Control (*n* = 3578)		Case (*n* = 2995)		*P*-value	Control (*n* = 14,026)		Case (*n* = 18,439)		*P*-value
	No.	%	No.	%		No.	%	No.	%		No.	%	No.	%	
Age (years)															
0–64	502	25.4	420	21.8	0.009	2304	64.39	1825	60.9	0.004	8449	60.2	9513	51.6	<0.0001
≥65	1475	74.6	1503	78.2		1274	35.61	1170	39.1		5577	39.8	8926	48.4	
Gender															
Male	1514	76.6	1520	79.0	0.06	2417	67.55	2093	69.9	0.04	8639	61.6	11,495	62.3	0.37
Female	463	23.4	403	21.0		1161	32.45	902	30.1		5384	38.4	6941	37.6	
Omitted											3	0.02	3	0.02	
Smoking status															
Never	633	32.0	138	7.1	<0.0001	867	24.23	137	4.6	<0.0001	4415	31.5	1800	9.8	<0.0001
Ever	1339	67.7	1774	92.3		2702	75.52	2854	95.3		9930	66.5	16,341	88.6	
Omitted	5	0.3	11	0.6		9	0.25	4	0.1		281	2.0	298	1.6	
Histology															
Squamous			488	25.4				307	10.3				4490	24.3	
Adenocarcinoma			788	40.9				620	20.7				6819	37.0	
Other			613	31.9				226	7.5				5487	29.8	
Omitted			34	1.8				1842	61.5				1643	8.9	

### Selection of index SNPs and candidate SNPs in discovery

To determine the most important susceptibility loci for lung cancer and to identify multiple association signals within observed loci, we performed association analyses using the 1st and 2nd discovery cohort, separately, and conducted a meta-analysis of the two cohorts in the discovery phase. We identified the most significant susceptibility loci for lung cancer on chromosomes 15q25.1 in both discovery cohorts, and confirmed the finding in the meta-analysis. Eight signals within chromosome 15q25.1 were defined as lung cancer risk-associated SNPs based on *P* values of association with lung cancer of less than 5 × 10^−8^ in the 1st discovery cohort and in the meta-analysis (Table 2). After Bonferroni correction, the eight signals maintained a significant impact on lung cancer risk in the 1st discovery cohort and in the meta-analysis. We defined the eight significant SNPs, which were rs1051730 in *CHRNA*3; rs1996371, rs6495314, rs11638372, rs4887077, and rs6495309 in *CHRNB4*; and rs8034191 and rs2036534 in *HYKK*, as the index SNPs for lung cancer risk, and used these eight SNPs to further select the candidate SNPs, which interacted with the eight index SNPs.

**Table 2:** Index SNPs in the chromosome 15q25.1 locus which were associated with lung cancer with *P* < 5.00E-8 in the 1st discovery cohort and in meta-analysis of the discovery cohorts

SNP	Gene	Predicted function	A1	A2	1^st^ discovery cohort		2^nd^ discovery cohort		Meta-analysis of discovery cohorts		replication cohort	
					*P*-value	BONF^a^	*P*-value	BONF^a^	*P*-value	BONF^a^	*P*-value	BONF^a^
rs1051730	CHRNA3	coding	T	C	2.28E-14	7.77E-11	3.03E-13	1.04E-09	1.64E-25	5.09E-20	3.11E-49	1.06E-45
rs1996371	CHRNB4	intronic	G	A	9.08E-12	3.10E-08	1.15E-05	3.93E-02	2.05E-14	6.36E-09	2.83E-24	9.65E-21
rs6495314	CHRNB4	intronic	C	A	1.47E-11	5.01E-08	7.29E-06	2.49E-02	1.47E-14	4.56E-09	8.54E-24	2.91E-20
rs8034191	HYKK	intronic	C	T	3.05E-11	1.04E-07	8.98E-14	3.07E-10	2.40E-23	7.45E-18	2.12E-46	7.23E-43
rs11638372	CHRNB4	intronic	T	C	3.14E-10	1.07E-06	2.95E-05	1.01E-01	8.11E-13	2.52E-07	5.28E-24	1.80E-20
rs2036534	HYKK	3downstream	C	T	3.81E-10	1.30E-06	4.29E-06	1.47E-02	7.81E-14	2.42E-08	4.85E-32	1.65E-28
rs4887077	CHRNB4	intronic	T	C	4.16E-10	1.42E-06	2.39E-05	8.17E-02	7.72E-13	2.40E-07	2.23E-23	7.61E-20
rs6495309	CHRNB4	3downstream	T	C	3.57E-08	1.22E-04	4.29E-06	1.47E-02	2.18E-12	6.76E-07	9.34E-29	3.19E-25

^a^*P*-value was adjusted for multiple comparisons using Bonferroni correction.

To evaluate potential functional connections between genes mapping throughout the genome and those on chromosome 15q25.1, to further elucidate the role of the chromosome 15q25.1-related pathway in lung cancer risk, we investigated SNP–SNP interactions between the eight index SNPs within chromosome 15q25.1 and the whole genome in both discovery cohorts, and conducted a meta-analysis of SNP–SNP interaction with both cohorts. A total of 5883 SNP pairs between the eight index SNPs and candidate SNPs in the whole genome exhibited epistasis *P* value of less than 0.05 in the 1st discovery cohort and in the meta-analysis results and showed epistasis *P* value of less than 0.10 in the 2nd discovery cohort (Supplementary Data 1). In total, 3409 candidate SNPs within the whole genome were identified and validated to interact with the eight index SNPs (Supplementary Data 2).

### Susceptibility pathways and GO terms in discovery

In order to identify chromosome 15q25.1-associated pathogenic pathways and biological processes that may be relevant to lung cancer etiology, we then conducted enrichment analyses using i-GSEA4GWAS^13^ in discovery phase with the meta-analysis results of 2530 SNPs, which were pruned from eight index SNPs and 3409 candidate SNPs for linkage disequilibrium (LD) to reduce the possibility of biased results. We applied mapping rules of SNPs to genes by incorporating a region 20 kb upstream and downstream of each gene (Supplementary Data 2 and 3). In total, one Kyoto Encyclopedia of Genes and Genomes (KEGG) pathway, three Reactome pathways, and 22 Gene Ontology (GO) terms were significantly associated with lung cancer risk with improved gene set enrichment analysis (i-GSEA)^13^ *P* values less than 0.05 and FDR less than 0.25 for each pathway (Table 3). The KEGG pathway was the neuroactive ligand receptor interaction pathway (i-GSEA *P* = 0.001 and FDR = 0.006). The 22 GO terms included substrate-specific channel activity (i-GSEA *P* < 0.001 and FDR = 0.005), ion channel activity (i-GSEA *P* < 0.001 and FDR = 0.005), gated channel activity (i-GSEA *P* = 0.002 and FDR = 0.006), and several similar terms.

**Table 3:** Pathways and GO terms in discovery and replication with a threshold of FDR < 0.25 in both phase

Source	Pathway/gene set name	Meta-analysis of discovery cohorts		Replication cohort	
		*P*-value	FDR	*P*-value	FDR
KEGG	neuroactive ligand receptor interaction	0.001	0.006	0.013	0.042
Reactome	neuronal system	0.001	0.015	0.014	0.082
	transmission across chemical synapses	0.003	0.023	0.003	0.028
Gene Oncology	substrate-specific channel activity	<0.001	0.005	0.002	0.004
	ion channel activity	<0.001	0.005	0.002	0.004
	substrate-specific transporter activity	0.001	0.006	0.010	0.013
	cation channel activity	0.002	0.006	0.002	0.008
	ion transmembrane transporter activity	0.002	0.006	0.004	0.009
	metal ion transmembrane transporter activity	0.001	0.006	0.002	0.003
	transmembrane transporter activity	<0.001	0.006	0.007	0.012
	gated channel activity	0.002	0.006	0.001	0.016
	substrate-specific transmembrane transporter activity	<0.001	0.006	0.006	0.012
	cation transmembrane transporter activity	0.001	0.006	0.003	0.006
	transmembrane receptor activity	0.001	0.007	<0.001	0.006
	receptor activity	0.017	0.021	0.002	0.007
	macromolecular complex	0.001	0.008	0.006	0.037
	protein complex	0.001	0.012	0.006	0.080
	intrinsic to membrane	0.003	0.022	0.002	0.025
	intrinsic to plasma membrane	0.004	0.024	0.002	0.030
	integral to membrane	0.003	0.027	0.002	0.027
	membrane part	0.005	0.028	0.013	0.050
	membrane	0.006	0.032	0.024	0.071
	plasma membrane part	0.009	0.032	0.005	0.030
	integral to plasma membrane	0.003	0.035	0.002	0.051
	plasma membrane	0.015	0.044	0.034	0.085

### Susceptibility pathways and GO terms in replication

We also examined whether our findings of chromosome 15q25.1-related pathways could be validated as involved in lung cancer pathogenesis and conducted an independent GWAS with a population-based case-control study among 18,439 lung cancer cases and 14,026 healthy controls in the replication phase using i-GSEA4GWAS. Of the eight index SNPs and the 3409 candidate SNPs in the discovery phase, 3411 SNPs were found in the replication cohort and, after pruned, 2525 SNPs were applied for enrichment (Supplementary Data 2). The replication cohort analysis confirmed that the eight index SNPs within chromosome 15q25.1 were significantly associated with lung cancer risk with a logistic regression *P* value of each index SNP of less than 1 × 10^−22^ (Table 2). Enrichment analysis in the replication cohort confirmed that the KEGG pathway, two Reactome pathways, and 22 GO terms were all significantly associated with lung cancer risk with *P* values of less than 0.05 and FDR of less than 0.25 for each pathway, which was in agreement with the findings from the meta-analysis of the discovery phase (Table 3).

### Verification of GWAS pathway analysis

Considering the possibility that the much more significant lung cancer associated *P* values in the index SNPs than in the candidate SNPs might lead to false positive enrichment if the observed pathways were due only to the significance of the index SNPs, we performed gene set enrichment analysis with the index SNPs alone and the candidate SNPs alone, separately, to clarify the contribution of the index SNPs alone and the candidate SNPs alone to pathway analysis. We found that the enrichment analysis with the index SNPs alone in discovery and replication, respectively, cannot result in any pathways and GO terms with threshold of FDR < 0.25, but the analysis with the candidate SNPs alone showed several pathways and GO terms with threshold of FDR < 0.25 in discovery and replication, respectively (Supplementary Table 2). To further elucidate the independent effect of the candidate SNPs on this pathway enrichment, we conducted an analysis using the observed logistic regression *P* values of the candidate SNPs and setting the *P* values of the index SNPs to 0.01 to reduce the impact that these SNPs might have had on the analysis. We observed that 14 significant GO terms, all of which are from the 22 susceptibility GO terms confirmed by the discovery and replication phase of GWAS analyses, were associated with lung cancer risk with i-GSEA *P* values of less than 0.05 and FDR of less than 0.25 for each pathway in both the discovery and replication data. Therefore, our sensitivity analyses deny the possibility that the observed pathways are due only to effects from only the index SNPs.

In order to demonstrate that the observed pathways were independent of the tool-chain, we performed gene set enrichment analysis using an alternative analytical strategy, namely GSA-SNP2^14,15^. We found that the KEGG pathway, all of the 22 GO terms and one Reactome pathways, which were observed from our previous GWAS analyses with i-GSEA4GWAS, showed significant association with lung cancer risk with *P* values of less than 0.05 for each pathway in both discovery and replication (Supplementary Table 3). Only one Reactome pathway, neuronal system pathway, from our previous GWAS analyses was unable to be confirmed. A few additional pathways, such as receptor complex term, were identified. In addition, this method provides more precise *P* values.

### Functional validation by lung eQTL analysis

We next measured genome-wide gene expression levels in lung tissues of 409 lung cancer patients and mapped eQTLs to determine which genes can be transcriptionally regulated by SNPs in chromosome 15q25.1, and asked whether genes on chromosome 15q25.1 and its related genes identified by eQTL studies would indicate shared pathways with the susceptibility pathways and GO terms from our GWA study. Because rs16969968 was a functional SNP that changes signal transduction through *CHRNA*5^16^, and since rs16969968 had an estimated *R*-square LD value of 0.98 with rs1051730, which was the most significant SNP associated with lung cancer risk in discovery and replication cohorts, we used rs16969968 as a surrogate for *CHRNA3*–*CHRNA5* and to investigate the influence of rs16969968 on whole-genome gene expression level. In addition, because rs6495309^17,18^ in *CHRNB4* and rs8034191^2,17^ in *HYKK* had been reported to exhibit the strongest association with lung cancer risk in *CHRNB4* and *HYKK*, separately, we also explored the effect of rs6495309 and rs8034191 on whole-genome gene expression level (Supplementary Data 4).

**Fig. 2:**
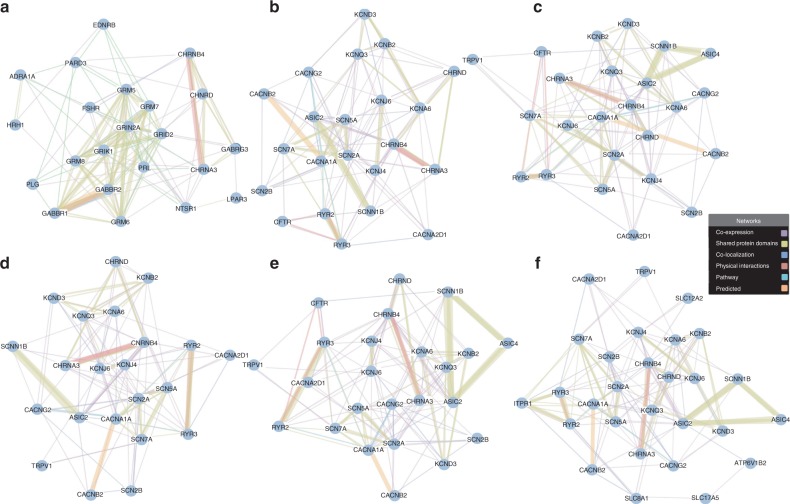
Gene network of the susceptibility pathways/GO terms. Each node is a gene, which was the selected gene in our GWAS pathway analysis and is shown in Supplementary Data 4. The connecting lines are drawn if the two genes have a relationship such as co-expression, shared protein domain, physical interactions, co-localization, or pathway. The thickness of the lines represents the degree of similarity between two genes. Gene network is produced using GeneMANIA. **a** The network of KEGG neuroactive ligand receptor interaction pathway consists of 23 genes and only TRPV1 in the associated gene list identified by our GWAS enrichment was shown no direct relationship with other associated genes. **b** The network of gated channel activity GO term consists all of 22 genes identified by our GWAS enrichment. **c** The network of ion channel activity GO term consists all of 24 genes identified by our GWAS enrichment. **d** The network of cation channel activity GO term consists all of 22 genes identified by our GWAS enrichment. **e** The network of substrate-specific channel activity GO term consists all of 24 genes identified by our GWAS enrichment. **f** The network of cation transmembrane transporter activity GO term consists 29 and only SLC4A4 in the associated gene list genes identified by our GWAS enrichment was shown no direct relationship with other associated genes

We evaluated whether the epistatistic pathways we previously identified were significantly related to expression levels. The KEGG neuroactive ligand receptor interaction pathway from our GWAS analyses was validated to influence lung cancer risk through an impact on expression levels. The GWAS pathways of Reactome showed no significant associations with lung cancer risk. Of 22 susceptibility GO terms from our GWAS analyses, gated channel activity was significantly associated with lung cancer risk (Fisher’s exact test, *P* = 0.029). Four transporter activity GO terms, including ion channel activity, cation channel activity, substrate-specific channel activity, and cation transmembrane transporter activity, had borderline significant associations with lung cancer (Fisher’s exact test, *P* = 0.071, 0.073, 0.080, and 0.098, respectively) (Table 4 and Fig. 2). Another 17 GO terms exhibited no significant relationship. We also evaluated whether the functional eQTL analysis identified the same lung cancer-related pathways after removing the HYKK and CHRNB4, which were eQTL related pathways of genes underlying rs16969968, rs6495309, and rs8034191.

**Table 4:** Functional annotation of eQTL study results for our susceptibility GWAS GO terms with a threshold of *P* value < 0.1

GO term	*P*-value
gated channel activity	0.029
ion channel activity	0.071
cation channel activity	0.073
substrate-specific channel activity	0.08
cation transmembrane transporter activity	0.098

We observed that the KEGG neuroactive ligand receptor interaction pathway still exhibited an involvement in lung cancer risk through its effect on expression levels. The gated channel activity term showed association with lung cancer risk with borderline significance (Fisher’s exact test, *P* = 0.088) (Supplementary Table 4).

**Fig. 3:**
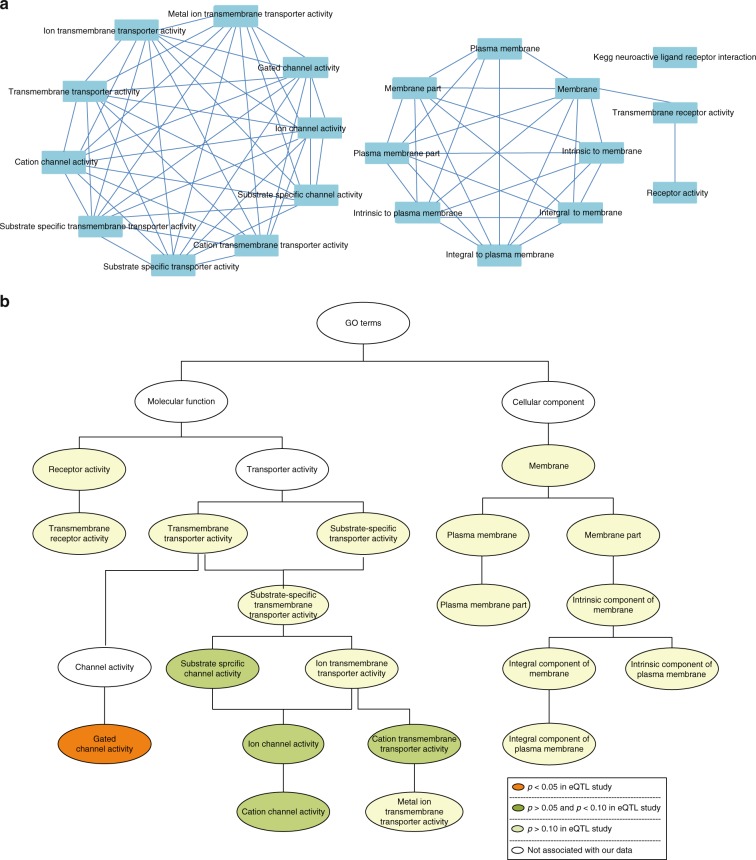
Association among susceptibility pathways and GO terms. **a** Each node is a subcomponent. Connecting lines are drawn if the overlapping coefficient between the two nodes is greater than 0.8. This picture was drawn by Cytoscape with EnrichmentMap plugin, using the standard gene set file. **b** The relationship between the susceptibility GO terms. The relationship between the GO terms was based on the knowledge from the European Bioinformatics Institute. All gene sets which were enriched in our GWAS pathway analyses were colored. Of the GO terms, 10 terms belonged to a transporter activity term. Both substrate-specific transporter activity term and transmembrane transporter activity term are part of the transporter activity term and have a child term of substrate-specific transmembrane transporter activity. Both terms of substrate-specific channel activity and ion transmembrane transporter activity are part of the substrate-specific transmembrane transporter activity term, and have a child term of ion channel activity which had a child term of cation channel activity. In addition, cation transmembrane transporter activity was part of ion transmembrane transporter activity, and had a child term of metal ion transmembrane transporter activity. Gated channel activity term was a child term of transmembrane transporter activity and shared several child terms with ion channel activity term

To aid interpretation of the relationship of the sharing pathways in both GWAS analysis and eQTL studies, we calculated the overlapping genes in the KEGG neuroactive ligand receptor interaction pathway and the GO terms and clarified the parent and child terms of the GO terms (Fig. 3). In addition, we investigated the gene expression level in normal lung tissue from Genecards database and found that all the genes in the neuroactive ligand receptor interaction pathway and the gated channel activity term, as well as the four transporter activity terms whose functional annotation for eQTL study had borderline significant association with lung cancer, are normally expressed in normal lung tissue and may play roles in cell growth, differentiation, or function of normal lung cell.

### Combined effect of genes on lung cancer risk

In order to explore whether genes from our susceptibility KEGG pathways and GO terms could jointly affect lung cancer risk, we calculated the individual and combined effects of multiple functionally-related genes from our susceptibility pathways and GO terms on lung cancer risk. Supplementary Data 5 shows the results from our study of all selected genes, which were identified by our GWAS enrichment analysis as significant genes constituting the susceptibility pathways/GO terms, and the reference SNP, as well as its *P* value associated with lung cancer risk. Since the combined effect of weaker SNPs/genes might have minor influence and lead to difficulties in exploring the systems view, only those genes whose reference SNPs were associated with lung cancer risk with border-line significance (association test *P* < 0.1) in the meta-analysis of the discovery cohorts and in the replication cohort were selected to assess the individual and joint effects on lung cancer risk. With the threshold of *P* value of the reference SNP less than 0.1, the same genes/SNPs were selected in gated channel activity term and the 4 transporter activity terms whose functional annotation for eQTL study were borderline significantly associated with lung cancer. Therefore, we explored the accumulated risk in the neuroactive ligand receptor interaction pathway and the gated channel activity term.

In total, for the neuroactive ligand receptor interaction pathway, *CHRNA*3 rs1051730 and *CHRNB4* rs6495309 reached the criterion and were included for further analysis of the independent association and combined effects of SNPs on lung cancer risk. With respect to the gated channel activity term, *CHRNA3* rs1051730, *CHRNB4* rs6495309, *KCNJ4* rs138396, and *SCN2B* rs7944321 reached the criterion and were included for further analysis. Because the frequency of *CHRNA3* rs1051730 T, *CHRNB4* rs6495309 C, *KCNJ4* rs138396 A, and *SCN2B* rs7944321 A alleles among the cases were slightly higher than among controls in the discovery cohorts and in the replication cohort, we assumed these alleles may be putative risk alleles in further combined analyses.

The association of lung cancer risk and genotypes of each SNP and the number of risk alleles is shown in Tables 5 and 6. In each cohort, the observed genotype frequencies among the controls were all consistent with Hardy–Weinberg equilibrium. Among the selected genes and their reference SNPs, *CHRNA3* rs1051730 was the most significantly associated with increased lung cancer risk. With respect to *CHRNA3* rs1051730, compared with the CC homozygote, the CT heterozygote was associated with an elevated risk of lung cancer with ORs being 1.32 (adjusted 95% CI, 1.21–1.44) in meta-analysis of the discovery cohorts and 1.27 (adjusted 95% CI, 1.21–1.33) in replication, while the TT homozygote was associated with increased lung cancer risk with ORs being 1.89 (adjusted 95% CI, 1.67–2.14) in meta-analysis of the discovery cohorts and 1.63 (adjusted 95% CI, 1.52–1.75) in replication. We also found and validated a significant dose–response relationship between the number of *CHRNA3* rs1051730 T alleles and lung cancer risk (adjusted trend test *P* *=* 2.68 × 10^−24^ for discovery (Table 5) and adjusted trend test *P* = 1.82 × 10^−44^ for replication (Table 6)). The risk allele of *CHRNB4* rs6495309 also significantly increased lung cancer risk in discovery and replication. A significant dose–response relationship was demonstrated between the number of risk alleles of CHRNB4 rs6495309 and the risk of lung cancer. Each of the other SNPs, including *KCNJ4* rs138396 and *SCN2B* rs7944321, appeared to have a slightly elevated risk of lung cancer in discovery and replication.

**Table 5:** Individual and combined effects of SNPs from our susceptibility pathways on lung cancer risk in the meta-analysis of Discovery Cohorts

		Univariate analysis					Multivariate analysis*				
		OR	L95	U95	*P*	*P*_trend	OR	L95	U95	*P*	*P*_trend
CHRNA3											
rs1051730	0	1				1.29E-25					2.68E-24
	1	1.31	1.21	1.43	3.46E-10		1.32	1.21	1.44	1.23E-09	
	2	1.86	1.65	2.09	8.80E-25		1.89	1.67	2.14	7.44E-24	
CHRNB4											
rs6495309	0	1				2.35E-12					4.56E-11
	1	1.22	1	1.5	0.05		1.21	0.98	1.49	0.08	
	2	1.58	1.3	1.93	5.21E-06		1.56	1.27	1.91	2.67E-05	
KCNJ4											
rs138396	0	1				0.06					0.04
	1	1	0.91	1.09	0.94		1.01	0.92	1.1	0.89	
	2	1.13	1.01	1.26	0.03		1.15	1.02	1.29	0.02	
SCN2B											
rs7944321	0	1				0.07					0.14
	1	1.07	0.98	1.16	0.13		1.06	0.97	1.15	0.19	
	2	1.12	0.94	1.34	0.22		1.09	0.91	1.32	0.35	
neuroactive ligand receptor interaction pathway											
(CHRNA3 rs1051730 and CHRNB4 rs6495309)											
	0-1	1				2.80E-26	1				1.55E-24
	2	1.32	1.18	1.47	8.45E-07		1.32	1.18	1.48	1.82E-06	
	3	1.48	1.32	1.65	4.94E-12		1.47	1.31	1.65	6.14E-11	
	4	2.04	1.79	2.33	2.22E-26		2.07	1.8	2.38	4.99E-25	
gated channel activity term											
(CHRNA3 rs1051730, CHRNB4 rs6495309, KCNJ4 rs138396 and SCN2B rs7944321)											
	0-1	1				4.39E-19					2.46E-18
	2-3	1.29	1.08	1.53	5.50E-03		1.3	1.08	1.56	5.50E-03	
	4-5	1.6	1.34	1.9	1.84E-07		1.63	1.36	1.96	1.65E-07	
	6-8	2.15	1.74	2.65	9.31E-13		2.17	1.74	2.7	4.01E-12	

*Adjusted by age sex smoke status in the Logistic Models.

**Table 6:** Individual and combined effects of SNPs from our susceptibility pathways on lung cancer risk in Replication Cohort

		Univariate analysis					Multivariate analysis*				
		OR	L95	U95	*P*	*P_trend*	OR	L95	U95	*P*	*P_trend*
CHRNA3											
rs1051730	0	1				1.70E-51	1				1.82E-44
	1	1.28	1.22	1.34	3.43E-23		1.27	1.21	1.33	2.62E-20	
	2	1.65	1.54	1.77	2.47E-46		1.63	1.52	1.75	1.22E-40	
CHRNB4											
rs6495309	0	1				1.40E-29	1				1.55E-24
	1	1.14	1.02	1.28	0.02		1.12	1	1.26	0.05	
	2	1.46	1.31	1.63	1.32E-11		1.42	1.27	1.6	2.13E-09	
KCNJ4											
rs138396	0	1				2.00E-04	1				5.00E-04
	1	1.06	1.01	1.11	0.03		1.05	1	1.11	0.08	
	2	1.13	1.06	1.21	2.00E-04		1.13	1.06	1.21	4.00E-04	
SCN2B											
rs7944321	0	1				8.90E-03	1				0.01
	1	1.06	1.01	1.11	0.026		1.05	1	1.11	0.04	
	2	1.1	0.99	1.22	0.083		1.1	0.99	1.23	0.08	
neuroactive ligand receptor interaction pathway											
(CHRNA3 rs1051730 and CHRNB4 rs6495309)											
	0-1	1				1.11E-58	1				4.80E-50
	2	1.21	1.14	1.28	1.79E-09		1.21	1.14	1.29	2.96E-09	
	3	1.44	1.36	1.54	1.44E-30		1.42	1.33	1.52	2.43E-26	
	4	1.77	1.64	1.91	2.65E-49		1.74	1.61	1.89	3.32E-43	
gated channel activity term											
(CHRNA3 rs1051730, CHRNB4 rs6495309, KCNJ4 rs138396 and SCN2B rs7944321)											
	0-1	1				3.36E-44	1				2.09E-37
	2-3	1.18	1.07	1.3	8.00E-04		1.15	1.04	1.28	5.40E-03	
	4-5	1.51	1.37	1.67	4.89E-17		1.47	1.33	1.63	7.77E-14	
	6-8	1.79	1.59	2.02	2.84E-22		1.72	1.52	1.95	8.27E-18	

*Adjusted by age sex smoke status in the Logistic Model

For the neuroactive ligand receptor interaction pathway, based on the number of risk alleles of the combined *CHRNA3* rs1051730 and *CHRNB4* rs6495309 genotypes, we grouped the individuals into four genotype groups, as follows: zero or one risk alleles of either gene; only two risk alleles; three risk alleles; and four risk alleles (Tables 5 and 6). We observed that the combined genotypes in those carrying four risk alleles, compared with those carrying zero or one risk allele, had a >2-fold increased risk in discovery (adjusted OR = 2.07; 95% CI, 1.80–2.38), and exhibited a 1.74-fold elevated risk in replication (adjusted OR = 1.74; 95% CI, 1.61–1.89) for lung cancer risk. The difference in *CHRNA3* rs1051730 and *CHRNB4* rs6495309 combination was associated with lung cancer risk in a dose-dependent fashion in discovery (adjusted trend test *P* = 1.55 × 10^−24^) and replication (adjusted trend test *P* = 4.80 × 10^−50^).

Based on the number of risk alleles of the combined genotypes in the gated channel activity term, we grouped the individuals into four genotype groups, as follows: zero or one risk allele of either gene; only two or three risk alleles; four or five risk alleles; and six to eight risk alleles (Tables 5 and 6). Compared with individuals with zero or one risk allele, we observed that the combined genotypes in those carrying six to eight risk alleles had a >2-fold increased risk in discovery (adjusted OR = 2.17; 95% CI, 1.74–2.70) and a 1.7-fold elevated risk in replication (adjusted OR = 1.72; 95% CI, 1.52–1.95) for lung cancer risk. The difference between the four genotype groups had a significant association with lung cancer risk in a dose-dependent fashion in discovery (adjusted trend test *P* = 2.46 × 10^−18^) and in replication (adjusted trend test *P* = 2.09 × 10^−37^).

### Stratified gene enrichment analyses by smoking status

When we performed the stratified analyses according to smoking status, we found that chromosome 15q25.1 was the most significant susceptibility locus for lung cancer risk among smokers in the 1st and 2nd discovery cohort, and a meta-analysis of discovery cohorts also supported this finding. Eight SNPs within chromosome 15q25.1 were identified and validated as associated with smoking-related lung cancer and were defined as the index SNPs for further selection of the candidate SNPs (Supplementary Table 5). In total, 3401 candidate SNPs (Supplementary Data 6) in the whole genome were identified and verified to interact with eight index SNPs in the 1st and 2nd discovery cohort and in the meta-analysis of discovery. After pruning for LD, we conducted enrichment analyses with 2522 SNPs (Supplementary Data 7). Among those SNPs that are significant at *P* < 0.05, pathway analysis found the same one KEGG pathway and eight GO terms were identified and validated as significantly associated with lung cancer^19^ risk in the meta-analysis of discovery cohorts and in the replication cohort with statistically significant *P* values (Supplementary Table 6). In addition, we found that the KEGG neuroactive ligand receptor interaction pathway exhibited a significant association with smoking-related lung cancer risk in the meta-analysis results of the discovery phase (i-GSEA *P* = 0.004 and FDR = 0.017) and in the replication cohort (i-GSEA *P* < 0.001 and FDR = 0.003). We did not explore chromosome 15q25.1-related for lung cancer in never smokers because the association between SNPs within chromosomes 15q25.1 and lung cancer did not reach genome-wide significance in the discovery cohorts.

## Discussion

The chromosome 15q25.1 locus was first identified as the leading susceptibility locus for lung cancer in Caucasians in 2008 by our group^2^ and by Hung et al.^3^, and was then replicated in a Chinese population^18^, in African-Americans^7,17^, and by an international lung cancer consortium^20^, as well as in smokers^21^. However, to our knowledge, no study to date has investigated how this locus affects lung cancer etiology, nor documented the susceptibility pathways by which chromosome 15q25.1 modifies lung cancer risk and is involved in lung cancer pathogenesis. The results presented here confirm the central role of chromosome 15q25.1 in lung cancer pathogenesis and provide confirmation of the pathways that affect lung cancer pathogenesis. We identified the neuroactive ligand receptor interaction pathway is involved as a mechanism by which the chromosome 15q25.1 locus influences lung cancer risk, in large discovery cohorts and in the replication cohort, and confirmed the involvement using functional annotation of an eQTL study with lung tissue from lung cancer patients. Gated channel activity term was verified to be significantly associated with the mechanism of chromosome 15q25.1 in conferring lung cancer risk in GWAS pathway analysis of discovery and replication phase and in the functional annotation of an eQTL study. In addition, risk alleles in SNPs in the genes in our susceptibility pathways can be combines to confer the lung cancer risk.

Pathway analyses, being a complementary approach to single-point analyses, can determine whether a set of genes from a biological pathway jointly affects the risk of a disease trait and uncover insights into disease etiology, and therefore such analyses are beneficial to better understand the bridge between genotypes and phenotypes. GWAS pathway analyses together with gene expression studies identified new pathways involved in the etiology of cardiovascular disease^22^, immune-related disorders^23,24^, and body fat distribution^25^. The first wave of GWA studies to explore lung cancer susceptibility regions identified several candidate genes and causal variants for lung cancer risk. Going forward, investigation of the pathogenic pathways will be essential to provide a better understanding of the process of lung cancer etiology and will contribute to further control of lung cancer.

The neuroactive ligand receptor interaction pathway mainly consists a group of neuroreceptor genes, such as dopamine receptor^26^ and proto-oncogene, and is involved in environmental information processing and signaling molecules and interaction^27^. This pathway was found to be associated with certain neuropsychiatric disorders and congenital diseases^28,29^. A recent study of 23 lung squamous cell carcinoma and paired normal lung tissue evaluated gene expression associated with microRNA-375 and found that the neuroactive ligand receptor interaction pathway was one of the possible pathways associated with lung squamous cell carcinoma^30^. Another study investigated the differentially expressed genes in 48 lung adenocarcinomas and 47 controls and revealed this pathway was one possible mechanism of lung adenocarcinoma^31^.

These two reports revealed the dysregulation of the neuroactive ligand receptor interaction pathway in lung cancer and supported our findings that this pathway plays a role in lung cancer etiology.

The neuroactive ligand receptor interaction pathway is also implicated in nicotine dependence, which also contributes to increasing lung cancer risk. Most of the selected genes of this pathway from the current GWAS pathway analyses, including, *CHRNA5*–*CHRNA3*–*CHRNB4*^32^, *GABBR1*^33^, *GABBR2*^33^, *GRM7*^34^, *GRM8*^35^, *GRIN2A*^35^, and *CHRND*^36^ are significantly associated with nicotine dependence and smoking behavior, as well as known smoking-related diseases such as lung cancer. For example, GRM7 in chromosome 3p26.1 and GRM8 in chromosome 7q31.33 are important in the biological processes and development of nicotine dependence, and some of these risks may be shared across diverse population^34,35^. Second, the neurotransmitter receptors in this pathway (including *CHRNA5*, *CHRNA3*, *CHRNB4*, and *CHRND*) participate in the biological process by which smoking induces nicotine dependence. Thus, the association between chromosome 15q25.1, this pathway, lung cancer likely reflects, at least partially, an indirect effect of these genes on lung cancer risk through their effects on smoking behavior. Aside from nicotine dependence and lung cancer risk, this pathway also influences other neurotransmitter-mediated disorders, such as alcohol dependence^37^, Parkinson’s disease^38^, schizophrenia drug therapy^39^, and autism spectrum disorders^40^. Thus this pathway may have many complex effects on lung cancer risk, either directly by influencing lung tissues or lung cancers as suggested by expression studies, indirectly through smoking behavior or even through effects on other neurotransmitter-related diseases.

Another important finding in our study is that a few transporter activity GO terms, such as gated channel activity, were implicated in the mechanisms of chromosome 15q25.1-modified lung cancer risk. Although we first reported the association between the transporter activity GO terms and lung cancer risk, this finding could be supported by previous studies. First, numerous studies have shown that a few transporter activity GO terms are involved in the processes driving the malignancy, such as calcium channels^41,42^, which belong to the gated channel group or ion channel group. Second, *CHRNA5*–*CHRNA3*–*CHRNB4* within chromosome 15q25.1 has been documented to modify some pathways of gated channel activity and ion channel activity, and therefore to play a crucial role in leading to and maintaining malignant phenotypes^43^. Finally, the majority of genes which were chosen as the significant or selected genes in the current GWAS pathway analyses, such as *KCNJ4*^44^, *CACNB2*^45^, and *SLC14A*^46^, have been reported to be involved in cancer etiology and development. In addition, our finding that genes from our susceptibility transporter activity GO terms jointly affected the chromosome 15q25.1-related lung cancer risk also supported the hypothesis that these pathways were implicated in the mechanisms of lung cancer. Therefore, we speculated that the accumulated effects of multiple functionally-related genes from our susceptibility pathways caused lung cancer occurrence, even though a single gene in any pathway may have only a moderate or weak effect on lung cancer risk. However, more biological mechanism research involving these pathways needs to be carried out in future.

Our GWAS pathway analyses also suggest that receptor activity GO terms and membrane terms, might play roles in the mechanism via which the chromosome 15q25.1 locus is involved in the pathogenesis of lung cancer, though the involvement exhibits nonsignificant association in the functional annotation of eQTL studies. This finding was supported by the fact that most selected genes in the two pathways from current GWAS pathway analyses, including *CHRNA3*, *CHRNB4*, *TGFBR2*^47^, *RTPRG*^48^, *FGFR1*^49^, *OPCML*^50,51^, and *ROR1*^52,53^, were involved in influencing lung cancer risk. It is thus likely that combined effects and interaction of the genes in the two susceptibility pathways triggered lung cancer pathogenesis. Although we do not know at this stage whether the biological pathways identified in our study have a direct functional role in affecting lung cancer etiology, the susceptibility pathways represent attractive candidates.

Despite these intriguing findings in this well-characterized pathway study, our investigation still had some limitations. First, we performed the epistasis test between SNPs in the univariate model; this may have led to the omission of the effects of other cofactors, such as age, gender, and smoking status, on the interaction between SNPs. However, not including cofactors typically reduces power rather than false positive findings, so that a model ignoring cofactors seems a reasonable first step to analysis. In addition, we only retained the SNP pairs, which exhibited statistically significant interactions in the 1st discovery cohort and in the meta-analysis results and showed at least a borderline significant interaction in the 2nd discovery cohort, for further analysis, which ensured the reliability of this study. Second, only GWAS data without genome-wide expression data were applied to identify the SNPs and their related genes that interact with the chromosome 15q25.1 locus. However, the susceptibility pathways and GO terms from our GWA study can share pathways with the functional annotation of genes in chromosome 15q25.1 and its related genes identified by eQTL studies, which supports the interpretation of some of our findings. A concern in this study is the large number of tests that were performed to identify epistatically acting SNPs. However, the purpose of conducting SNP–SNP interaction test in the current study is to select a group of candidate SNPs which are the most associated with the index SNPs in chromosome 15q25.1. On the other hand, majority of SNPs/genes in the pathways have weak and minor influence on the pathogenesis of complex disorder^12^. Therefore, we applied a pathway-based approach to identify the sets of pathways that were significantly associated with cancer risk. To correct for multiple testing associated with pathway analysis we followed a false discovery rate approach. Identification and verification of the susceptibility pathways in both GWAS analysis of discovery and replication and the eQTL study confirmed the reliability of our study. Nevertheless, our results should be confirmed in the future with genome-wide expression data and protein–protein interaction data, and more biological mechanism research involving these pathways needs to be carried out. Finally, we realized that, in the GWAS pathway analyses, all subjects used in both discovery and replication phase are of European ancestry, and that the subjects in the lung eQTL study are French Canadians, which suggest that our findings can be applied to the population of European ancestry.

Many genetic variants certainly contribute to the large unexplained portion of lung cancer pathogenesis, and it is expected that more mechanisms contributing to increased lung cancer risk will be identified in the future. The data presented here suggest that common genetic variations within chromosome 15q25.1 are likely to affect lung cancer etiology by influencing the expression/structure and thereby the function of genes that comprise the neuroactive ligand receptor interaction pathway or gated channel activity and related terms. To the best of our knowledge, this is the first study to explore the pathogenic pathways related to the mechanisms through which the chromosome 15q25.1 locus modifies lung cancer risk. These pathways provide important leads to a better understanding of the etiology and development of lung cancer, potentially shorten the interval between biologic knowledge and improved patient care, and are beneficial to the design of future functional studies to increase understanding of these mechanisms.

## Methods

### Study subjects

The study design is presented in Fig. 1. In the discovery phase, two discovery cohorts were used, to perform SNP selection and GWAS pathway analysis. The Environment And Genetics in Lung cancer Etiology (EAGLE) study^54^, which was composed of 1923 lung cancer cases and 1977 healthy controls, was used as the 1st discovery cohort. The EAGLE study participants were recruited in Italy between 2002 and 2005 for a population-based case-control study, which included incident primary lung cancer cases of any histologic type and healthy population-based controls, matched by gender, residence, and 5-year age-group. All subjects in the EAGLE study are of Italian nationality and born in Italy.

We used the M.D. Anderson Cancer Center (MDACC) study^2^ and the International Agency for Research on Cancer (IARC) study^55^ as the second discovery cohort, in total comprising 2995 lung cancer cases and 3578 healthy controls. The MDACC study participants were recruited at the University of Texas MD Anderson Cancer Center between 1997 and 2007 and included 1154 primary lung cancer cases of adenocarcinoma and squamous cell carcinoma and 1136 healthy controls that were matched to cases by smoking behavior, ethnicity, and 5-year age-group. The IARC study was a multicenter study from six countries of central Europe, which recruited newly-diagnosed lung cancer cases of any histologic type and healthy individuals without diagnosed cancers or any family history of cancers, matched to cases by sex, age, and center or region within European countries. The current case-control comparison included 1841 cases and 2442 controls from IARC available data. All subjects used in the current study are of European ancestry.

The Oncoarray consortium, which analyzed samples of 18,439 European-descent lung cancer cases and 14,026 European-descent healthy controls, was used for replication. The Oncoarray consortium is a network created to increase understanding of the genetic architecture of common cancers and included GWAS data of a total of 57,776 samples, obtained from 29 studies across North America and Europe, as well as Asia^56^. The participants who lacked imputed data, disease status, were close relatives (second-degree relatives or closer) or had low-quality DNA, or were non-European, were excluded from the current study. Therefore, a total of 18,439 cases and 14,026 healthy controls were included in the current case-control study.

Noncancerous lung tissue from Laval University was obtained from 420 patients undergoing surgical resection for lung cancer. Through quality controls, 409 samples were used for whole-genome gene expression profiling in the lung and eQTL analysis. All patients in the Laval cohort were from a French Canadian population and underwent lung cancer surgery between April 2004 and December 2008. Samples were stored at the Institut universitaire de cardiologie et de pneumologie de Quebec (IUCPQ) site of the Respiratory Health Network Tissue Bank of the Fonds de la recherche en sante du Quebec (www.tissuebank.ca)^57^. Lung tissue samples were obtained in accordance with Institutional Review Board guidelines. Genotype data and a detailed pathology reports were available for all patients.

Human participant approval was obtained from the Institutional Review Board of each participating Hospital and University and by the National Cancer Institute, Bethesda, MD, USA. Written informed consent was obtained from each participant.

### Genotyping

A total of 561,466 SNPs in EAGLE samples were genotyped using Illumina HumanHap550v3_B BeadChips (Illumina, San Diego, CA, USA) at the Center for Inherited Disease Research, part of the Gene Environment Association Studies Initiative (GENEVA) funded through the National Human Genome Research Institute. Genotyping of 317,498 SNPs in MDACC samples was carried out using Illumina 300K HumanHap v1.1^55^. A further 317,139 SNPs in IARC samples were genotyped using either Illumina 317k or 370Duo arrays^55^. A novel technology developed by Illumina to facilitate efficient genotyping was used to genotype a total of 494,763 SNPs in Oncoarray samples^56^. rs16969968 in the lung eQTL study was genotyped using the Illumina Human1M-Duo BeadChip^10^.

### Imputation

To effectively replicate the findings in the discovery phase, we imputed additional SNPs in Oncoarray samples to allow us to integrate the data with the common SNPs studied in the discovery cohorts. Imputation was performed with the software package Impute 2 v2.3.2^58^ and 1000 Genomes Project Phase 3 (ftp://ftp.1000genomes.ebi.ac.uk/vol1/ftp/phase3/data). Following imputation, 20,734,083 SNPs in the whole genome were available for further analysis.

### Association analysis and meta-analysis

Case-control association tests for genotyped data were conducted using 1-degree-of-freedom Cochran–Mantel–Haenszel tests with the application of PLINK version 1.9. SNPTEST v2.5.2 was used in the analysis of case-control association for each SNP in imputed data. Meta-analysis of the 1st and 2nd discovery cohorts was performed on the results of case-control association analysis using the basic meta-analysis function in PLINK v1.9, which conducted a fixed-effects analysis using inverse variance weighting to combine the studies. In the discovery phase, a total of 310,276 SNPs were the same in both the 1st and 2nd discovery cohorts, passed quality control steps and were retained for association analyses and meta-analyses.

### Index SNP selection

SNPs within the 15q25.1 locus spanning 203 kb, which were associated with lung cancer risk with *P* values of less than 5 × 10^−8^ in the 1st discovery cohort and in the meta-analysis of the 1st and 2nd discovery cohort, were selected as index SNPs. A Bonferroni correction was applied to adjust the association of the index SNP for multiple comparisons with using PLINK version 1.9 and R package of adjust *P*-values for multiple comparisons. Because 310,276 SNPs in the discovery phase were performed for association analyses and meta-analyses and 3411 SNPs in the replication phase were conducted association analyses, adjustments for 310,276 tests in discovery and 3411 tests in replication were used. After selection, eight SNPs met the criterion for index SNP selection and were used for further selection of the candidate SNPs in the whole genome which had potential functional connections with the index SNPs in the chromosome 15q25.1 locus.

### Epistasis test and candidate SNP selection

The epistasis test between SNPs in the whole genome and the chromosome 15q25.1 locus was performed separately for the 1st and 2nd discovery cohorts using the application PLINK version 1.9. A total of 2,482,200 SNP × SNP pairs were calculated in both cohorts. We then carried out a meta-analysis to combine the epistasis results in the 1st and 2nd discovery cohorts with the application of the basic meta-analysis function in PLINK v1.9 that conducted fixed-effects analysis using inverse variance weighting.

The SNPs, which interacted with the index SNPs within the 15q25.1 locus with an epistasis *P* value of less than 0.05 in the 1st discovery cohort and in the meta-analysis of both discovery cohorts, and less than 0.10 in the 2nd discovery cohort, were selected as the candidate SNPs for further pathway analyses. After selection, 3409 candidate SNPs met the criterion for candidate SNP selection and were identified to have potential connections with the eight index SNPs in the chromosome 15q25.1 locus.

### Pathway analysis with GWAS data

We included curated pathways from the Canonical pathways, Reactome, BioCarta, KEGG databases^59^, and GO^60^. The Reactome database is based on reactions between diverse molecular species rather than limiting the pathways to protein–protein interactions. The KEGG database represents experimentally-validated pathways of metabolic processes and gene sets of human diseases. GO is a major framework for the model of biology that defines classes used to describe gene function, and relationships between these concepts.

Gene set enrichment analysis was performed by i-GSEA4GWAS. SNPs were retained for analysis that were within 20 kb upstream or downstream of a gene. We used gene set databases of canonical pathways, GO biological process, GO molecular function, and GO cellular component, separately, and applied the standard input gene set file of KEGG, BioCarta and, Reactome, which were downloaded from the Molecular Signatures Database (MSigDB) in GSEA (http://software.broadinstitute.org/gsea/msigdb/collections.jsp), we selected gene sets whose number of genes were between 21 and 200, and without limiting gene sets by keyword (e.g. immune) and without masking the MHC region. In order to reduce the possibility of biased results due to LD patterns from SNP arrays^13^, we pruned the set of SNPs, including the index SNPs and the candidate SNPs, for LD and only inputted SNPs not in LD (*r*^2^ < 0.2) to enrich pathways. After pruning, we performed gene set enrichment analysis with associated *P* values of the SNPs that using the option of “−logarithm transformation”, as required by the software, in the meta-analysis of discovery cohorts and the replication cohort, respectively.

i-GSEA4GWAS performs gene set enrichment to identify pathways that show a higher proportion of statistically significant genes than randomly expected and, with some modifications, is based on the GSEA algorithm^61,62^. i-GSEA4GWAS implements SNP label permutation to analyze SNP *P* values and to correct gene and gene set variation and multiplies a significance proportion ratio factor to the enrichment score (ES) to yield the significant proportion-based enrichment score (SPES). SPES multiplies by the proportion of significant SNPs in the pathway so that i-GSEA4GWAS identifies pathways/gene sets including a high proportion of significant genes. It is, therefore, more appropriate for study of the combined effects of possibly modest SNPs/genes and gives i-GSEA improved sensitivity for complex diseases^13^. Pathways/gene sets with FDR < 0.25 were regarded as possibly associated with traits; FDR < 0.05 were regarded as high confidence or with statistical significance.

Gene set enrichment analysis was also performed by GSA-SNP2, which is a successor of GSA-SNP^14,15^, using same SNPs and *P* value and to retain SNPs with 20 kb upstream or downstream of a gene. We used gene set databases which were downloaded from the Molecular Signatures Database (MSigDB) in GSEA and selected gene sets whose number of genes was between 21 and 200.

### Genome-wide gene expression levels and eQTL study

All lung samples were reviewed by an experienced pathologist for clinical diagnosis and staging. Each lung tissue sample was snap-frozen in liquid nitrogen and stored at −80 °C until further processing. The SV96 Total RNA Isolation System (Promega) was used to extract RNA. Expression profiling was carried out with an Affymetrix custom array (GEO platform GPL10379). The robust multichip average method^63^ as implemented in the Affymetrix Power Tools software was used to examine expression values. Standard quality control parameters^64^ were applied to check the quality of the arrays. Through quality controls, 409 patients were available for eQTL analyses with both genotypes and gene expression levels. R statistical software was used to perform tests for robust multichip average expression. Association tests between the expression traits, which were adjusted for age, sex, and smoking status, and the most significant SNPs in each genes within chromosome 15q25.1 associated with lung cancer risk in previous reports, including rs16969968^16^, rs6495309^17,18^, and rs8034191^2,17^, were estimated with the application of quantitative association tests implemented in PLINK. A *P* value of less than 0.05 was considered to be significant.

### Functional validation of pathways with eQTL results

The genes in the whole genome with a linear regression *P* value of less than 0.0005 in the eQTL study were selected as candidate genes for further pathway/gene set analysis. Because the “Functional annotation table” of DAVID may query associated terms for all genes and “Functional annotation clustering” of DAVID can cluster functionally similar genes into groups^65^, we employed the “Functional annotation table” of DAVID (version 6.8) to perform functional annotation of biological pathways in Reactome, BioCarta, and KEGG and used “Functional annotation clustering” of DAVID (version 6.8) to cluster functionally similar genes into groups of GO terms, with Species and background being set up as Homo sapiens. We used the candidate genes in the whole genome with and without the genes in the chromosome 15q25.1 locus to perform the analyses, by choosing ‘Homo sapiens’ selection to limit annotations by species.

### Relationship of the susceptibility pathways and GO terms

We clarified the relationship of sharing pathways identified by GWAS analysis of both discovery and replication phase and validated by eQTL studies with information from the European Bioinformatics Institute (http://www.ebi.ac.uk/QuickGO/) and applied Cytoscape (version 3.4.0) with EnrichmentMap plugin (version 2.1). The standard gene sets for EnrichmentMap plugin were downloaded from MSigDB Collections (http://software.broadinstitute.org/gsea/msigdb/collections.jsp). For the selected genes in our susceptibility pathways from current GWAS pathway analyses, we achieved the gene expression level in normal lung tissue from Genecards database (http://www.genecards.org/).

### Accumulating risk of lung cancer

We calculated the individual and combined effect of genes in the pathways/gene set on lung cancer risk using the SNPs that were identified by i-GSEA4GWAS as reference SNPs for the selected genes in each pathway. The genes whose reference SNPs were associated with lung cancer risk with borderline significance (*P* < 0.1) in the meta-analysis of discovery cohorts and in the replication cohort, were selected to assess the individual effect and joint effects on lung cancer risk. Genotype frequencies between the cases and controls were evaluated using a chi-square test. Univariate and multivariate logistic regression models were used to calculate odds ratios (ORs) and 95% confidence intervals (CIs) of each genotype to estimate its effect on lung cancer risk with or without adjustment for age, sex and smoking status (never and ever). Statistical analyses were performed with Statistical Analysis System (SAS) software (version 9.1; SAS Institute, Cary, NC, USA) and *P* value < 0.05 was considered significant.

### Stratified analyses

We determined whether there were different pathways among the overall group, and in the group when stratified by smoking status (never and ever). In the subgroups of smokers and non-smokers, we used a similar process to select index SNPs and candidate SNPs, and to carry out pathway analyses. Among smokers, eight index SNPs and the 3401 candidate SNPs were selected for further gene set enrichment analysis in the 1st and 2nd discovery cohort, the meta-analysis of discovery cohorts and the replication cohort. Among non-smokers, no SNPs in chromosome 15q25.1 reached the criteria for index SNP selection, and therefore no subsequent steps for pathway analyses were conducted.

### Data availability

The data that support the findings of this study are available. The access numbers are “phs000336.v1.p1.c1” for EAGLE study, “phs000753.v1.p1” for MDACC study, and “phs001273” for Oncoarray study in dbGAP. The IARC study was made available at http://www.ceph.fr/cancer^3^.

## Electronic supplementary material

Supplementary Information

Description of Additional Supplementary Files

Supplementary Data 1

Supplementary Data 2

Supplementary Data 3

Supplementary Data 4

Supplementary Data 5

Supplementary Data 6

Supplementary Data 7
