# Supplementary material for: Identification of susceptibility pathways for the role of chromosome 15q25.1 in modifying lung cancer risk
Source: Nat Commun. 2018 Aug 13;9:3221. doi: 10.1038/s41467-018-05074-y (PMC6089967; doi:10.1038/s41467-018-05074-y)
Supplement: Supplementary file 1 — Supplementary Information [file 41467_2018_5074_MOESM1_ESM.pdf]

# Identification of susceptibility pathways for the role of chromosome 15q25.1 in modifying lung cancer risk

Ji et al.

**Supplementary table 1**. Participant characteristics for the eQTL study

| Variants | All participants (n = 420) | |
| --- | --- | --- |
| | No. | % |
| Age (years) | 63.4± 10.0 | |
| | | |
| Gender | | |
| Male | 235 | 55.95 |
| Female | 185 | 44.05 |
| | | |
| Smoking Status | | |
| Never | 37 | 8.81 |
| Ever | 383 | 91.19 |
| | | |
| Histology | | |
| Squamous | 240 | 57.14 |
| Adenocarcinoma | 108 | 25.72 |
| Other | 72 | 17.14 |

**Supplementary table 2**. Pathways and GO terms in sensitivity analysis for pathway enrichment with threshold of FDR < 0.25 in either phase of discovery and replication

| | | Enrichment with actual P values of the candidate SNPs alone | | | | Enrichment with using the observed P values of the candidate SNPs and setting the P values of the index SNPs to 0.01 | | | |
|---|---|---|---|---|---|---|---|---|---|
| | | Meta-analysis of Discovery Cohorts | | Replication Cohort | | Meta-analysis of Discovery | | Replication Cohort | |
| Source | Pathway/Gene set name | P-value | FDR | P-value | FDR | P-value | FDR | P-value | FDR |
| KEGG | neuroactive ligand receptor interaction | | | | | 0.004 | 0.024 | | |
| Reactome | neuronal system | | | | | 0.004 | 0.09 | | |
| | transmission across chemical synapses | | | | | 0.018 | 0.086 | | |
| | immune system | 0.021 | 0.1245 | | | 0.044 | 0.139 | | |
| | transmembrane transport of small molecules | 0.016 | 0.214 | | | 0.017 | 0.111 | | |
| | gpcr downstream signaling | | | | | | | 0.039 | 0.217 |
| Gene Oncology | substrate specific channel activity | | | | | 0.002 | 0.025 | 0.004 | 0.017 |
| | ion channel activity | | | | | 0.002 | 0.025 | 0.004 | 0.017 |
| | gated channel activity | | | | | 0.016 | 0.024 | 0.003 | 0.052 |
| | cation channel activity | | | | | 0.009 | 0.022 | 0.003 | 0.032 |
| | ion transmembrane transporter activity | | | | | 0.005 | 0.019 | 0.024 | 0.057 |
| | metal ion transmembrane transporter activity | | | | | 0.003 | 0.019 | 0.007 | 0.023 |
| | transmembrane transporter activity | | | | | 0.002 | 0.012 | 0.04 | 0.071 |
| | substrate specific transmembrane transporter activity | | | | | 0.002 | 0.015 | 0.036 | 0.073 |
| | cation transmembrane transporter activity | | | | | 0.004 | 0.013 | 0.014 | 0.041 |
| | transmembrane receptor activity | | | 0.022 | 0.24 | 0.01 | 0.022 | 0.002 | 0.021 |
| | receptor activity | | | | | 0.042 | 0.075 | 0.01 | 0.038 |
| | protein complex | | | | | 0.003 | 0.058 | 0.046 | 0.191 |
| | intrinsic to membrane | 0.045 | 0.184 | | | 0.018 | 0.087 | 0.039 | 0.227 |
| | membrane part | | | | | 0.019 | 0.064 | 0.042 | 0.196 |
| | substrate specific transporter activity | | | | | 0.004 | 0.012 | | |
| | macromolecular complex | | | | | 0.003 | 0.039 | | |
| | intrinsic to plasma membrane | | | | | 0.014 | 0.073 | | |
| | integral to membrane | 0.037 | 0.1904 | | | 0.013 | 0.098 | | |
| | membrane | | | | | 0.02 | 0.081 | | |
| | plasma membrane part | | | | | 0.024 | 0.083 | | |
| | integral to plasma membrane | | | | | 0.009 | 0.07 | | |

| | | | | | | | | | |
|---|---|---|---|---|---|---|---|---|---|
| | intracellular organelle part | 0.042 | 0.227 | | | 0.038 | 0.082 | | |
| | organelle part | 0.042 | 0.227 | | | 0.038 | 0.082 | | |
| | phosphotransferase activity alcohol group as acceptor | | | 0.036 | 0.19 | | | 0.049 | 0.072 |
| | protein kinase activity | | | | | | | 0.033 | 0.068 |
| canonical pathways | mark signaling pathway | | | 0.042 | 0.08 | | | 0.03 | 0.07 |

**Supplementary table 3**. Pathways and GO terms in discovery and replication with threshold of P value < 0.05 in both phase using GSA-SNP2

| | Pathway/Gene set name | Meta-analysis of Discovery Cohorts p-value | Replication Cohort p-value |
|---|---|---|---|
| KEGG | neuroactive ligand receptor interaction | 4.71E-06 | 5.11E-08 |
| Reactome | neurotransmitter receptor binding and downstream transmission in the postsynaptic cell | 1.51E-05 | 4.39E-11 |
| | transmission across chemical synapses | 3.45E-04 | 8.87E-09 |
| | adaptive immune system | 3.92E-02 | 4.08E-03 |
| | l1cam interactions | 4.82E-02 | 1.59E-02 |
| Gene Oncology | substrate specific transporter activity | 1.36E-06 | 1.11E-04 |
| | transmembrane transporter activity | 3.12E-06 | 6.17E-05 |
| | cation transmembrane transporter activity | 3.13E-06 | 4.22E-07 |
| | substrate specific transmembrane transporter activity | 5.77E-06 | 1.15E-05 |
| | ion transmembrane transporter activity | 1.21E-05 | 3.16E-06 |
| | cation channel activity | 2.23E-05 | 4.94E-10 |
| | metal ion transmembrane transporter activity | 4.15E-05 | 8.36E-09 |
| | ion channel activity | 7.26E-05 | 6.28E-10 |
| | substrate specific channel activity | 7.26E-05 | 6.28E-10 |
| | gated channel activity | 1.46E-04 | 1.13E-10 |
| | transmembrane receptor activity | 2.78E-03 | 1.31E-07 |
| | receptor activity | 1.11E-02 | 1.50E-06 |
| | voltage gated cation channel activity | 2.74E-02 | 9.57E-04 |
| | voltage gated channel activity | 2.74E-02 | 9.57E-04 |
| | receptor complex | 6.35E-04 | 5.32E-11 |
| | integral to membrane | 1.22E-03 | 1.85E-03 |
| | integral to plasma membrane | 1.45E-03 | 7.55E-04 |
| | intrinsic to membrane | 1.57E-03 | 1.88E-03 |
| | intrinsic to plasma membrane | 1.95E-03 | 7.90E-04 |
| | membrane part | 2.17E-03 | 6.56E-03 |
| | plasma membrane part | 3.35E-03 | 5.74E-04 |
| | protein complex | 9.28E-03 | 2.13E-04 |
| | macromolecular complex | 1.90E-02 | 1.36E-03 |
| | plasma membrane | 2.36E-02 | 3.65E-03 |
| | membrane | 2.51E-02 | 1.54E-02 |

**Supplementary table 4**. Functional annotation of eQTL study results for our susceptibility GWAS GO terms

| | | Analysis with genes in chromosome 15q25.1 and its related genes | Analysis with the related genes alone |
|---|---|---|---|
| Category | GO Term | *P* -value | *P* -value |
| GO:0022836 | gated channel activity | 0.029 | 0.088 |
| GO:0005216 | ion channel activity | 0.071 | 0.17 |
| GO:0005261 | cation channel activity | 0.073 | 0.2 |
| GO:0022838 | substrate-specific channel activity | 0.08 | 0.186 |
| GO:0008324 | cation transmembrane transporter activity | 0.098 | 0.199 |
| GO:0015075 | ion transmembrane transporter activity | 0.128 | 0.229 |
| GO:0022891 | substrate-specific transmembrane transporter activity | 0.221 | 0.348 |
| GO:0022857 | transmembrane transporter activity | 0.282 | 0.42 |
| GO:0005886 | plasma membrane | 0.354 | 0.391 |
| GO:0022892 | substrate-specific transporter activity | 0.37 | 0.514 |
| GO:0046873 | metal ion transmembrane transporter activity | 0.371 | 0.358 |
| GO:0005887 | integral to plasma membrane | 0.59 | 0.708 |
| GO:0031226 | intrinsic to plasma membrane | 0.636 | 0.747 |
| GO:0004888 | transmembrane signaling receptor activity | 0.676 | 0.798 |
| GO:0044459 | plasma membrane part | 0.695 | 0.77 |
| GO:0004872 | receptor activity | 0.775 | 0.862 |
| GO:0031224 | intrinsic to membrane | 0.852 | 0.872 |
| GO:0016020 | membrane | 0.865 | 0.866 |
| GO:0016021 | integral to membrane | 0.876 | 0.895 |
| GO:0044425 | membrane part | 0.961 | 0.967 |

**Supplementary table 5**. Index SNPs in the chromosome 15q25.1 locus which were associated with lung cancer among smokers

| SNP | Gene | Predicted function | Position | A1 | A2 | 1st discovery cohort | | | 2nd discovery cohort | | | Meta-analysis of Discovery Cohorts | | | Replication Cohort | | |
|---|---|---|---|---|---|---|---|---|---|---|---|---|---|---|---|---|---|
| | | | | | | *P* -value | OR | BONF* | *P* -value | OR | BONF* | *P* -value | OR | BONF* | *P* -value | OR | BONF* |
| rs1051730 | CHRNA3 | coding | 76681394 | T | C | 3.10E-14 | 1.49 | 1.06E-10 | 1.19E-11 | 1.31 | 4.04E-08 | 2.05E-23 | 1.37 | 6.36E-18 | 2.71E-52 | 1.34 | 9.24E-49 |
| rs8034191 | HYKK | intronic | 76593078 | C | T | 2.29E-11 | 1.42 | 7.79E-08 | 1.60E-11 | 1.30 | 5.46E-08 | 5.32E-21 | 1.34 | 1.65E-15 | 3.99E-51 | 1.33 | 1.36E-47 |
| rs1996371 | CHRNB4 | intronic | 76743861 | G | A | 3.34E-11 | 1.41 | 1.14E-07 | 2.96E-04 | 1.15 | 1.00E+00 | 6.77E-12 | 1.24 | 2.10E-06 | 3.12E-24 | 1.21 | 1.06E-20 |
| rs6495314 | CHRNB4 | intronic | 76747584 | C | A | 7.40E-11 | 1.40 | 2.52E-07 | 1.73E-04 | 1.16 | 1.00E+00 | 5.05E-12 | 1.24 | 1.57E-06 | 1.69E-23 | 1.21 | 5.76E-20 |
| rs11638372 | CHRNB4 | intronic | 76770614 | T | C | 8.48E-10 | 1.37 | 2.89E-06 | 4.10E-04 | 1.15 | 1.00E+00 | 8.37E-11 | 1.22 | 2.60E-05 | 3.28E-23 | 1.20 | 1.12E-19 |
| rs4887077 | CHRNB4 | intronic | 76765419 | T | C | 1.15E-09 | 1.37 | 3.92E-06 | 3.97E-04 | 1.15 | 5.90E-01 | 9.96E-11 | 1.22 | 3.09E-05 | 2.93E-23 | 1.20 | 9.99E-20 |
| rs2036534 | HYKK | 3downstream | 76614003 | C | T | 2.02E-09 | 0.69 | 6.90E-06 | 1.22E-04 | 0.83 | 4.15E-01 | 2.46E-11 | 0.78 | 7.63E-06 | 1.69E-33 | 0.76 | 5.76E-30 |
| rs6495309 | CHRNB4 | 3downstream | 76702300 | T | C | 3.74E-08 | 0.71 | 1.27E-04 | 3.66E-05 | 0.82 | 1.25E-01 | 3.55E-11 | 0.78 | 1.10E-05 | 5.72E-29 | 0.78 | 1.95E-25 |

**P* -value was adjusted for multiple comparisons using Bonferroni correction.

**Supplementary Table 6.** Pathways and GO terms for smoking-related lung cancer in the discovery and replication cohorts with threshold of FDR < 0.25

| | | Meta-analysis of Discovery Cohorts | | Replication Cohort | |
|---|---|---|---|---|---|
| Source | Pathway/Gene set name | P-value | FDR | P-value | FDR |
| Kegg | neuroactive ligand receptor interaction | 0.004 | 0.017 | < 0.001 | 0.003 |
| Gene Oncology | receptor activity | 0.009 | 0.078 | < 0.001 | 0.019 |
| | substrate-specific channel activity | 0.009 | 0.098 | 0.002 | 0.012 |
| | metal ion transmembrane transporter activity | 0.022 | 0.103 | 0.002 | 0.018 |
| | cation transmembrane transporter activity | 0.033 | 0.119 | 0.005 | 0.011 |
| | ion channel activity | 0.008 | 0.122 | 0.002 | 0.01 |
| | transmembrane transporter activity | 0.035 | 0.133 | 0.006 | 0.022 |
| | transmembrane receptor activity | 0.001 | 0.144 | 0.002 | 0.015 |
| | cation channel activity | 0.006 | 0.146 | 0.002 | 0.011 |
