# Supplementary material for: Identification of susceptibility pathways for the role of chromosome 15q25.1 in modifying lung cancer risk
Source: Nat Commun. 2018 Aug 13;9:3221. doi: 10.1038/s41467-018-05074-y (PMC6089967; doi:10.1038/s41467-018-05074-y)
Supplement: Supplementary file 2 — Description of Additional Supplementary Files [file 41467_2018_5074_MOESM2_ESM.pdf]

Description of Additional Supplementary Files

File Name: Supplementary Data 1
Description: 5,883 SNP pairs which show significant interaction between the index SNPs in chromosome 15q25.1 and their interacting candidate SNPs in the discovery cohorts

File Name: Supplementary Data 2
Description: Summary of the index SNPs within chromosome 15q25.1 and the 3,409 candidate SNPs within whole genome

File Name: Supplementary Data 3
Description: The LD of the left 2530 SNPs after pruned from 8 index and 3409 candidated SNPs

File Name: Supplementary Data 4
Description: The eQTL results of the selected SNPs and genes in whole genome with threshold of P value < 0.0005.

File Name: Supplementary Data 5
Description: The selected genes and their reference SNPs of our susceptibility pathways and GO terms in GWAS enrichment analysis.

File Name: Supplementary Data 6
Description: The summary of the index SNPs within chromosome 15q25.1 and the 3,401 candidate SNPs within whole genome for smoking-related lung cancer

File Name: Supplementary Data 7
Description: The LD of the left index and candidated SNPs after pruned for smoking-related lung cancer.
